# A computationally tractable birth-death model that combines phylogenetic and epidemiological data

**DOI:** 10.1371/journal.pcbi.1009805

**Published:** 2022-02-11

**Authors:** Alexander Eugene Zarebski, Louis du Plessis, Kris Varun Parag, Oliver George Pybus

**Affiliations:** 1 Department of Zoology, University of Oxford, Oxford, United Kingdom; 2 MRC Centre for Global Infectious Disease Analysis, Imperial College London, London, United Kingdom; Johns Hopkins University, UNITED STATES

## Abstract

Inferring the dynamics of pathogen transmission during an outbreak is an important problem in infectious disease epidemiology. In mathematical epidemiology, estimates are often informed by time series of confirmed cases, while in phylodynamics genetic sequences of the pathogen, sampled through time, are the primary data source. Each type of data provides different, and potentially complementary, insight. Recent studies have recognised that combining data sources can improve estimates of the transmission rate and the number of infected individuals. However, inference methods are typically highly specialised and field-specific and are either computationally prohibitive or require intensive simulation, limiting their real-time utility. We present a novel birth-death phylogenetic model and derive a tractable analytic approximation of its likelihood, the computational complexity of which is linear in the size of the dataset. This approach combines epidemiological and phylodynamic data to produce estimates of key parameters of transmission dynamics and the unobserved prevalence. Using simulated data, we show (a) that the approximation agrees well with existing methods, (b) validate the claim of linear complexity and (c) explore robustness to model misspecification. This approximation facilitates inference on large datasets, which is increasingly important as large genomic sequence datasets become commonplace.

## Author summary

Mathematical epidemiologists typically study time series of cases, ie the epidemic curve, to understand the spread of pathogens. Genetic epidemiologists study similar problems but do so using observations of the genetic sequence of the pathogen, which also contains information about the transmission process. There have been many attempts to unite these approaches and utilise both data sources. However, striking a suitable balance between model flexibility and fidelity, in a computationally tractable way, has proven challenging. There are several competing methods, but they are often intractable when applied to a large dataset. As sequencing of pathogen genomes becomes common, and an increasing amount of epidemiological data are collected, this situation will only be exacerbated. To bridge the gap between the time series and genomic methods we developed an approximation scheme, called TimTam. TimTam can accurately and efficiently estimate key features of an epidemic, such as the prevalence of infection (how many people are currently infected) and the basic reproduction number (a measure of the transmissibility of the infection.).

This is a *PLOS Computational Biology* Methods paper.

## Introduction

Estimating the prevalence of infection and transmission dynamics of an outbreak are central objectives of both infectious disease epidemiology and phylodynamics. In mathematical epidemiology, a time series of reported infections (known as the epidemic curve) is combined with epidemiological models to infer key parameters, such as the basic reproduction number, $R_{0}$, which is a fundamental descriptor of transmission potential [1, 2]. In phylodynamics, as applied to infectious disease epidemiology, phylogenies reconstructed from pathogen genetic sequences sampled over the course of an outbreak are used to estimate the size and/or growth rate of the infected population [3, 4].

Combining data from multiple sources has the potential to improve estimates of transmission rates and prevalence [5–7]. However, doing so raises substantial challenges. For example, there are technical difficulties associated with appropriately specifying joint distributions of multiple data sources and the resulting models may be complex and present computational challenges [8, 9]. In part due to this difficulty, phylogenetic and epidemiological inference methods have been developed and examined largely in isolation of each other [10, 11].

The two main frameworks for phylodynamic inference are the phylogenetic birth-death (BD) model, which estimates the *rate* of spread of the pathogen [12, 13] and the coalescent process, which estimates the *effective size* of the infected population [14, 15]. Within the coalescent framework, a phylogeny reconstructed from sampled sequences is related to the effective size of the infected population, assuming the sampled proportion of the population is small [14]. This relationship, when interpreted under a suitable dynamical model, allows the inference of epidemic dynamics [16, 17]. Both deterministic and stochastic epidemic models have been fitted to sequence data, providing estimates of prevalence and $R_{0}$ [17–19]. Considering the association between effective population size and time-varying covariates provides an additional way to model effective population sizes [20]. Combining sequence data with an epidemic time series allows inference of the epidemic size and its growth parameters [5]. However, early attempts to do so [5, 21] required the epidemic time series to be treated as independent of the sequence data, an approximation which only holds when the number of sequences is small relative to the outbreak size. Previously, coalescent models have neglected the informativeness of sequence sampling times, although recent work has found estimates of the effective size can be improved substantially by incorporating sampling times [22, 23].

In the BD framework, births represent transmission events and deaths represent cessation of being infectious, eg due to death, isolation or recovery [24]. The birth-death process was extended to model serially-sampled sequences as another type of death event [25]. Further extensions linked the BD process to a stochastic epidemic (SIR) model under strong simplifying assumptions [26]. The resulting model improved estimates of $R_{0}$ and provided the first means of inferring the number of unsampled members of the infected population (via estimates of epidemic prevalence). Deterministic SIR models have also been used in both the BD [27] and coalescent frameworks [17].

Particle-filter based methods allow for flexible modelling of both sequence and epidemic time series data [28, 29], and have enabled the inclusion of both population structure [21] and superspreading [30] into epidemiological analysis. While particle methods provide a comprehensive approach to fusing epidemiological and phylogenetic data, they are computationally intractable, relying on intensive simulation, which can limit their application. Data augmentation also provides a powerful approach to the inference problem, but again relies on intensive simulation [31].

Recently, progress has been made on developing numerical schemes for computing the likelihood of both sequence and time series data, thereby facilitating equivalent estimation as mentioned above [32, 33]. These methods have smaller computational overheads, but still require calculations that have a quadratic computational complexity, ie grow with the square of the size of the dataset. Moreover, the approximation used can be numerically unstable under certain conditions [34].

To the best of our knowledge, there is currently no existing phylogenetic inference method, in either the BD or coalescent frameworks, that can (i) formally combine both epidemiological and sequence data, (ii) estimate the prevalence of infection and growth rate, and (iii) be applied practically to large datasets. As sequencing costs continue to decline and large genome sequence datasets collected over the course of an outbreak become the norm, the need for a tractable solution to these problems grows [35]. Here we present the first steps towards such a solution by approximating, and then modifying, an existing approach [32].

In this manuscript we describe a novel birth-death-sampling model tailored for use in estimating the basic reproduction number and prevalence of infection in an epidemic. We start by reviewing existing sampling models for birth-death processes and derive a missing sampling model which has a natural interpretation in epidemiology, where data are usually only available in the form of binned (eg weekly) counts. For example, if a health care provider is unable to report new cases over the weekend one might expect an aggregated number of cases to be reported at the start of the following week. This is in contrast to sequence data, which are regularly reported with individual dates and modelled as having unique, exact sampling dates.

With several simulation studies we demonstrate empirically that our approximation (a) agrees with the output of an existing numerical scheme, (b) has linear complexity, considerably improving on existing computational approaches, which grow (approximately) quadratically with the size of the dataset, and (c) even with aggregated (binned) data, key parameters can still be recovered. Finally, we discuss the practical applications and benefits of TimTam and the limitations of our approach.

## Methods

Birth-death-sampling models are used to describe sequence data that have been either collected at predetermined points in time, hereafter *scheduled observations*, or opportunistically, ie when cases have presented themselves, hereafter *unscheduled observations* [13, 25]. The relationship between these sequences is described by the reconstructed phylogeny. An additional data type, sometimes referred to as *occurrence data* [29, 32], represents unscheduled observation of infectious individuals without their inclusion in the reconstructed phylogeny. Such occurrence data may arise, for example, when an individual tests positive for infection but the pathogen genome is not sequenced.

We categorise observations based on two attributes, (i) whether the infected individuals were observed at predetermined times (scheduled observations) or follow a point process (unscheduled observations), and (ii) whether the observed cases were included in the reconstructed phylogeny (a *sequenced* observation), or not (an *unsequenced* observation).

This categorisation suggests an additional data type: the time series of cases regularly studied in (non-genomic) epidemiology. In our terminology this is the scheduled observation of unsequenced cases, the removal of multiple individuals from the infectious population at the same time, without incorporating them into the reconstructed phylogeny. There are several benefits to being able to incorporate such data. First, since epidemiological data are often given as a time series (instead of a point process) this is arguably a more natural way to utilise occurrence data in the estimation process [36]. The same could be said for the sequenced samples in instances when multiple samples are collected on the same day [23]. The second benefit is computational; modelling observations as scheduled rather than unscheduled simplifies the likelihood, because a single scheduled observation can account for multiple unscheduled observations. As far as we are aware, scheduled unsequenced observations have not been considered in any phylodynamic inference method. Below we describe the sampling model formally and the method, TimTam, used to approximate the resulting likelihood. An implementation of this method is available from https://github.com/aezarebski/timtam and the version used here has been archived with the DOI https://doi.org/10.5281/zenodo.5761941.

### Phylogenetic birth-death process

**Fig 1:**
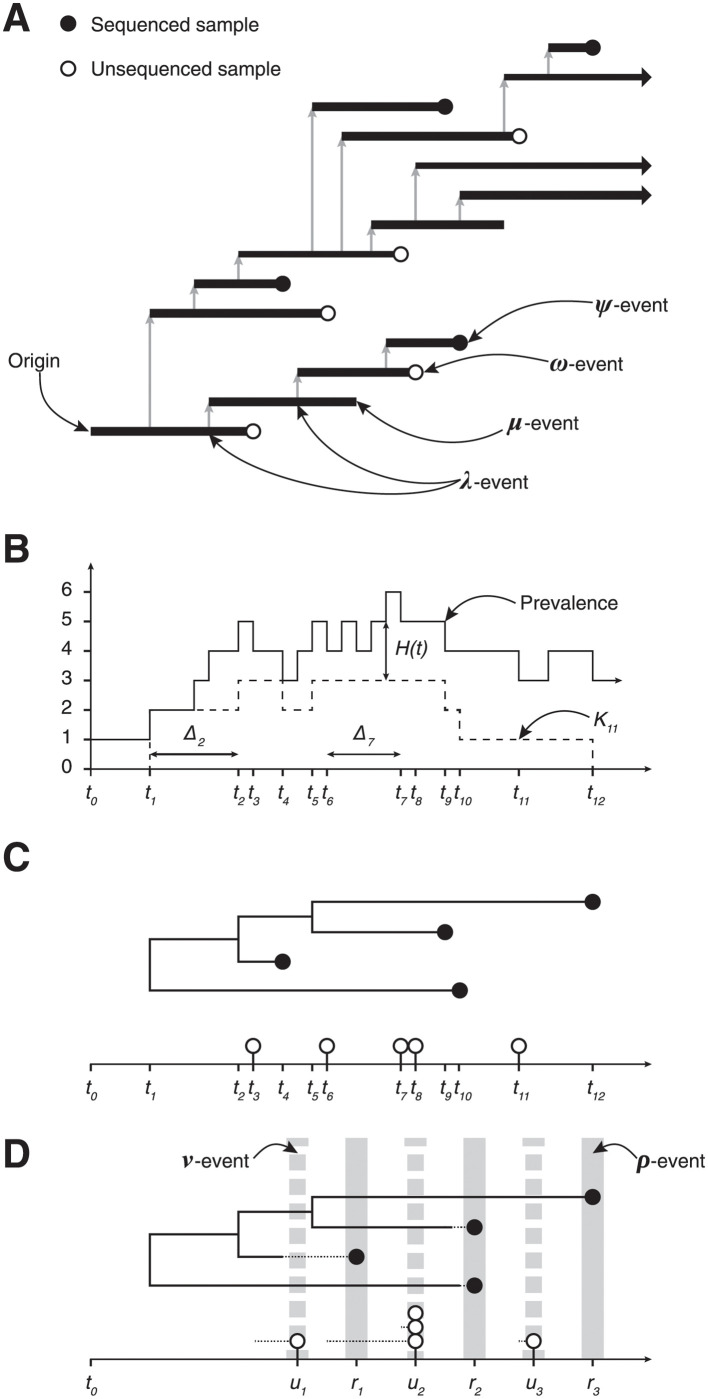
Birth-death model of transmission and observation. The process can be observed in several ways leading to different data types. **(A)** The transmission process produces a binary tree (the transmission tree) where an infection corresponds to a λ-event and a branch node and ceasing to be infectious corresponds to a *μ*-, *ψ*- or *ω*-event and a leaf node. **(B)** The number of lineages in the transmission tree through time, ie the prevalence of infection, and the number of lineages in the reconstructed tree, known as the lineages through time (LTT) plot, *K*_*i*_. **(C)** The tree reconstructed from the sequenced samples: *ψ*-events. The pathogen sequences allow the phylogeny connecting the infections and the timing of λ-events to be inferred. The unsequenced, *ω*-events form the point process on the horizontal axis. **(D)** Multiple *ψ*-events can be aggregated into a single *ρ*-event, such as the one at time *r*_2_. This loses information due to the discretization of the observation time, indicated by the dashed line segment. The same approach is used to aggregate *ω*-events into a single *ν*-event, eg the observation made at time *u*_2_.

The birth-death (BD) process starts with a single infectious individual at the time of origin, *t* = 0. Infectious individuals “give birth” to new infectious individuals at rate λ, and are removed from the process either through naturally ceasing to be infectious (at rate *μ*, often called the “death” rate), or through being sampled. There are two types of sampling in this model: scheduled and unscheduled and both can occur in a single realisation of the process. Unscheduled sampling of infectious individuals occurs at different rates depending on whether the samples are sequenced (which occurs at rate *ψ*) or not (which occurs at rate *ω*). An illustrative example of this process is shown in Fig 1A. Individuals can also be removed during *scheduled* sampling events (which are also referred to as *contemporaneous* sampling [13, 37], *concerted* sampling [27] and, informally, as *ρ*-sampling). A scheduled sampling event occurs at a predetermined time, during which each infectious individual is independently sampled with a fixed probability: during a sequenced scheduled sampling event each infectious individual is sampled (and sequenced) with probability *ρ*, and during an unsequenced scheduled sampling event each individual is sampled with probability *ν*. An illustrative example of the process, demonstrating the simultaneous use of both scheduled and unscheduled sampling, is shown in Fig A in S1 Appendix. We denote scheduled sampling times *r*_*i*_ for sequenced sampling and *u*_*i*_ for unsequenced sampling, and assume these times are known *a priori*, since they are under the control of those observing the system.

Realisations of the process are binary trees with internal nodes corresponding to infection events and terminal nodes representing removal events as shown in Fig 1 and Fig A in S1 Appendix. We assume the edges of the tree are labelled with their length to ensure the nodes appear at the correct depth. The tree containing all infected individuals is the *transmission tree* (Fig 1A). The subtree containing only the terminal nodes corresponding to sequenced samples (both scheduled and unscheduled) is called the *reconstructed tree* [12], (Fig 1C). In practice, the topology and branch lengths of the reconstructed tree are estimated from the pathogen genomes; here we assume these are known *a priori*.

Trees can be summarised by their *lineages through time* (LTT) plot, which describes the number of lineages in the tree at each point in time. We denote the number of lineages in the reconstructed tree at time *t*_*i*_ by *K*_*i*_ (Fig 1B). We define the number of *hidden* lineages through time as the number of lineages that appear in the transmission tree but not in the reconstructed tree. The number of hidden lineages at time *t* is denoted *H*(*t*), and for convenience as *H*_*i*_ at time *t*_*i*_. The types of data that we consider can be thought of as a sequence of *N* events, ${ℰ}_{1:N}$, starting from the origin and moving forward in time up to the present (ie the time of the last observation): ${ℰ}_{1:N}=\{(\Delta t_{i},e_{i},\Delta K_{i},\Delta H_{i}){\}}_{i=1\ldots N}$ with Δ*t*_*i*_ denoting the time since the previous observation (ie Δ*t*_*i*_ ≔ *t*_*i*_ − *t*_*i*−1_) and *e*_*i*_ describing the event that was observed at that time: *e*_*i*_ ∈ {λ-event, *ψ*-event, *ρ*-event, *ω*-event, *ν*- event}. The changes in the LTT and number of hidden lineages at time *t*_*i*_ are denoted Δ*K*_*i*_, so *K*_*i*_ = *K*_*i*−1_ − Δ*K*_*i*_, and Δ*H*_*i*_, so $H(t_{i})=H(t_{i}^{-})-\Delta H_{i}$. We use the left limit, $H(t_{i}^{-})$, because Δ*H*_*i*_ is the number of hidden lineages removed at time *t*_*i*_.

There are three important assumptions in the description above. The first is that once an individual has been sampled they are removed from the infectious population. This is a standard, though not universal, assumption and often justified by the fact that sampling broadly coincides with receiving medical care, and hence taking care not to spread the infection further. The second is that if there is a scheduled sample, it contains either all sequenced samples or all unsequenced samples, ie there are no scheduled samples with both sequenced and unsequenced observations. The observation model described above for unscheduled data has two potential interpretations: sequenced and unsequenced samples are selected completely independently, or alternatively, one could consider a two step process, in which we select samples and then decide whether to sequence them. Moving between these interpretations only requires simple adjustments to *ψ* and *ω*. However, unless we commit to one of these interpretations it is unclear how to extend the scheduled sampling model to admit mixtures of sequenced and unsequenced samples. Both for mathematical convenience and to maintain flexibility of the model we ignore mixed samples in this work. The third is that we assume the rate parameters are constant. Should variable rates be required to appropriately model changes in the observation process this could be achieved using established methods from the phylodynamic literature: skyline [15], skyride [38], etc.

### The likelihood

The joint conditional distribution of the process parameters, *θ* = (λ, *μ*, *ψ*, *ρ*, *ω*, *ν*), and the number of hidden lineages at time *t*_*N*_, *H*(*t*_*N*_), factorises as follows:

$$\begin{array}{c} f\left(\theta ,H_{N}|E_{1:N}\right)\propto f\left(H_{N}|E_{1:N},\theta \right)\underset{\text{Likelihood}}{\underset{︸}{f(E_{1:N}|\theta )}}\underset{\text{Prior}}{\underset{︸}{\pi (\theta )}}, \end{array}$$

where $f(H_{N}|{ℰ}_{1:N},\theta )$ is the posterior distribution of the prevalence given *θ* which can be used to obtain the posterior predictive distribution of the prevalence: $f(H_{N}|{ℰ}_{1:N})$. The likelihood has a natural factorisation which corresponds to processing the data from the origin through to the present:

$$\begin{array}{c} f\left(E_{1:N}|\theta \right)=\prod_{i=1}^{N}f\left(E_{i}|E_{1:(i-1)},\theta \right)=\prod_{i=1}^{N}c_{i}l_{i}. \end{array}$$ (1)

Since the likelihood of each observation depends on the distribution of the number of hidden lineages, the distribution of ${ℰ}_{i}$ depends on the whole history ${ℰ}_{1:(i-1)}$. Each factor, $f({ℰ}_{i}|{ℰ}_{1:(i-1)},\theta )$, can be expressed as a product, *c*_*i*_*l*_*i*_, where *c*_*i*_ is the probability that no events were observed during the interval of time, (*t*_*i*−1_, *t*_*i*_), and *l*_*i*_ is the probability that the event observed at the end of the interval is *e*_*i*_.

Let *M*(*t*, *z*) be the generating function (GF), in the variable *z*, for the distribution of *H*(*t*) and the observations up until time *t*:

$$\begin{array}{c} M\left(t,z\right)≔\sum_{h}P\left(H\left(t\right)=h,E_{1:x}:t_{x}\leq t\right)z^{h}. \end{array}$$

Note that because *M*(*t*, *z*) also accounts for the likelihood of the observations up until time *t* it is not necessarily a probability generating function (PGF), however it can be normalised to obtain a PGF: *M*(*t*, *z*)/*M*(*t*, 1) is a PGF. We make use of this property to calculate the likelihood by iterating over the observed events, ${ℰ}_{1:N}$, and keeping track of the normalisation constants, *M*(*t*_*i*_, 1).

Consider a sequence of functions, *M*_*i*_(*t*, *z*), which correspond to *M*(*t*, *z*) over the intervals (*t*_*i*_, *t*_*i*+1_), up to a normalisation constant which ensures *M*_*i*_(*t*_*i*_, 1) = 1. We define the *M*_*i*_ using a system of partial differential equations (PDEs). These equations are derived from the Master equations that describe how the number of hidden lineages changes through time.

$$\begin{array}{c} \begin{array}{cc} M_{i}\left(t_{i},z\right) & =F_{i}\left(z\right)\\ \partial_{t}M_{i} & =\left(\lambda z^{2}-\gamma z+\mu \right)\partial_{z}M_{i}+K_{i}\left(2\lambda z-\gamma \right)M_{i}, \end{array} \end{array}$$ (2)

where *γ* = λ + *μ* + *ψ* + *ω* and ∂_*x*_ is used to indicate partial differentiation with respect to the variable *x*. The number of lineages in the reconstructed tree, *K*_*i*_, only changes when there is a birth, or a sequenced sample and so is a constant over each interval.

The process starts with a single infected individual, so initially there are no hidden lineages and consequently the initial condition on the first interval is *M*_0_(0, *z*) = 1. Subsequent boundary conditions, *F*_*i*_(*z*), are based on the solution over the previous interval, *M*_*i*−1_ and the event that was observed at time *t*_*i*_.

The solution to Eq (2), first given as Proposition 4.1 in [32], is

$$\begin{array}{c} M_{i}\left(t,z\right)=F_{i}(p_{0}\left(t_{i+1}-t,z\right)){\left(\frac{p_{1}\left(t_{i+1}-t,z\right)}{1-z}\right)}^{K_{i}}. \end{array}$$ (3)

The functions *p*_0_ and *p*_1_ are standard results [25] describing the probability of an individual and their descendants giving rise to exactly zero or one observation during an interval of duration *t*_*i*+1_ − *t*; see S1 Appendix for further details.

Using Eq (3) the probability of not observing anything between times *t*_*i*_ and *t*_*i*+1_, and the probability generating function for the number of hidden lineages just prior to the observation at *t*_*i*+1_ are

$$\begin{array}{c} c_{i+1}=M_{i}\left(t_{i+1},1\right)\;\text{and}\;M_{i}\left(z\right)≔M_{i}\left(t_{i+1},z\right)/c_{i+1}. \end{array}$$ (4)

The process of calculating *l*_*i*+1_, the likelihood of observing ${ℰ}_{i+1}$, and the next boundary condition, *F*_*i*+1_(*z*), the PGF of the number of hidden lineages at *t*_*i*+1_ is carried out in two steps. First, we transform $M_{i}$ to account for the observation of ${ℰ}_{i+1}$ and evaluate the resulting expression at *z* = 1 to obtain *l*_*i*+1_ (using the transformations described below in Eqs (5), (6), (7) and (8)). Second, we normalise the coefficients of this GF to get the PGF of *H*(*t*_*i*+1_), which is the boundary condition, *F*_*i*+1_(*z*), in the PDE for *M*_*i*+1_ in Eq (2). This process is repeated for each interval of time to get all the *c*_*i*_ and *l*_*i*_ in Eq (1).

We will now describe the transformations to $M_{i}$ used to account for the observation of ${ℰ}_{i+1}$. Since λ- and *ψ*-events are only observed upon the reconstructed tree and do not influence the number of hidden lineages, $M_{i}$ is left unchanged when these are observed,

$$\begin{array}{c} \begin{array}{cc} l_{i+1} & =\{\begin{array}{cc} \lambda & E_{i+1}\;\text{is}\;\text{a}\;\lambda \text{-event}\\ \psi & E_{i+1}\;\text{is}\;\text{a}\;\psi \text{-event} \end{array}\\ F_{i+1}\left(z\right) & =M_{i}\left(z\right). \end{array} \end{array}$$ (5)

For an *ω*-event we need to shift the whole distribution of *H* and account for the unknown number of hidden lineages that could have been sampled, this is achieved by taking the partial derivative of the GF, which we denote by ∂_*z*_, as elaborated upon in S1 Appendix. The likelihood of an *ω*-event is the normalising constant after the differentiation:

$$\begin{array}{c} \begin{array}{cc} l_{i+1} & =\omega \partial_{z}M_{i}{\left(z\right)|}_{z=1},\\ F_{i+1}\left(z\right) & =\frac{\omega }{l_{i+1}}\partial_{z}M_{i}\left(z\right). \end{array} \end{array}$$ (6)

For a scheduled sampling event, at time *r*_*i*+1_ with removal probability *ρ*, we need to account for the survival of each of the *H*-lineages that were not sampled, those that were, and the number of lineages in the reconstructed tree that were not removed during this scheduled sampling. This leads to the following likelihood factor and updated PGF:

$$\begin{array}{c} \begin{array}{cc} l_{i+1} & =\frac{{\left(1-\rho \right)}^{K_{i+1}}\rho^{\Delta K_{i+1}}}{(\Delta K_{i+1})!}M_{i}\left(1-\rho \right),\\ F_{i+1}\left(z\right) & =\frac{{\left(1-\rho \right)}^{K_{i+1}}\rho^{\Delta K_{i+1}}}{\left(\Delta K_{i+1}\right)!l_{i+1}}M_{i}\left(\left(1-\rho \right)z\right). \end{array} \end{array}$$ (7)

The factor of 1 − *ρ* in the argument of $M_{i}$ is to account for the *H*-lineages that were not sampled. The factors of ${\left(1-\rho \right)}^{K_{i+1}}$ and $\rho^{\Delta K_{i+1}}$ come from the lineages in the reconstructed tree that were not sampled (of which there are *K*_*i*+1_), and those that were sampled (of which there are Δ*K*_*i*+1_).

Last, we include scheduled unsequenced samples, ie the observation and simultaneous removal of multiple lineages without subsequent inclusion in the reconstructed phylogeny. For Eq (6), we noted that a single *ω*-sampling event corresponds to differentiating the PGF of *H* once. If at time *t*_*i*+ 1_ there is a scheduled unsequenced sample where each infectious individual is sampled with probability *ν*, and *n* lineages in total are sampled, then we must take the *n*-th derivative and accumulate a likelihood factor for the removed and non-removed lineages of (1 − *ν*)^*K*^*ν*^*n*^ (assuming the LTT at that time is *K*). We also have to scale *z* by a factor of 1 − *ν* to account for the *H*-lineages that were not sampled. Therefore, as in Eqs (6) and (7), the likelihood and updated PGF after a *ν*-sample are:

$$\begin{array}{c} \begin{array}{cc} l_{i+1} & =\frac{{\left(1-\nu \right)}^{K_{i+1}}\nu^{\Delta H_{i+1}}}{(\Delta H_{i+1})!}\partial_{\hat{z}}^{\Delta H_{i+1}}M_{i}\left(\hat{z}\right){|}_{\hat{z}=\left(1-\nu \right)}\\ F_{i+1}\left(z\right) & =\frac{{\left(1-\nu \right)}^{K_{i+1}}\nu^{\Delta H_{i+1}}}{\left(\Delta H_{i+1}\right)!l_{i+1}}\partial_{\hat{z}}^{\Delta H_{i+1}}M_{i}\left(\hat{z}\right){|}_{\hat{z}=\left(1-\nu \right)z}, \end{array} \end{array}$$ (8)

where the use of $\hat{z}$ has been used to make explicit the order of operations.

Evaluating the expressions above numerically typically requires truncating a system of ordinary differential equations (ODEs) and solving them on each interval. This operation has a complexity which is cubic in the size of the truncated system (as a matrix exponential is required). Manceau *et al* [32] derived an approximation which has a quadratic complexity, albeit by introducing a further approximation. Our TimTam approximation, the main contribution of this paper, is as accurate as existing methods and has only a linear complexity.

### An analytic approximation

The **t**ime-series **i**ntegration **m**ethod **t**hrough **a**pproximation of **m**oments (TimTam) can be described as simply replacing the PGF of *H* with a more convenient PGF which describes a random variable with the same mean and variance. Specifically, we use the negative binomial (NB) distribution. We note two facts: first, we can evaluate the full PGF point-wise described above and, second, as shown in S1 Appendix, the GF of the negative binomial (NB) distribution is closed (up to a simple multiplicative factor) under partial derivatives and scaling of the parameter *z*. Together, these mean we can construct a NB approximation of the PGF at any point in the process and hence evaluate the resulting approximate likelihood and the distribution of hidden lineages. Algorithmically, this method can be expressed in the following steps:

1. Start at time *t*_*i*_ with the PGF *M*_*i*_ and use Eq (3) to obtain *M*_*i*_ at time *t*_*i*+1_.
2. Calculate *c*_*i*_ = *M*_*i*_(*t*_*i*+1_, 1^−^), the probability of not observing any events during the interval (*t*_*i*_, *t*_*i*+1_).
3. Define the PGF $M_{i}=M_{i}/c_{i}$ and the PGF resulting from approximating it with a NB distribution: ${\tilde{M}}_{i}$.
4. Use ${\tilde{M}}_{i}$ to compute, *l*_*i*_, the likelihood of observing ${ℰ}_{i+1}$ and let *M*_*i*+1_ be the PGF of the number of *H*-lineages conditioning upon this observation (see Eqs (6), (7) and (8)).
5. Increment the log-likelihood by log (*c*_*i*_*l*_*i*_) and return to Step 1 with an incremented *i* if there are remaining observations.

The steps involved require only the evaluation of closed form expressions and the number of iterations is linear with the number of observed events.

Our use of a NB moment-matching approximation is not arbitrary. Early work established the number of lineages descending from a single lineage has a zero-inflated geometric distribution [24], and the sum of independent and identically distributed geometric random variables follows a NB distribution. Our approach of treating the number of lineages derived from *n* individuals as a NB random variable is somewhat motivated by combining these two properties. Further support for our approximation is obtained by considering an equivalent BD process, but with the modified total birth rate of *λn* + *a* where *a* is a small offset representing an immigration rate that leads to the removal of the extra (unobservable) zeros. Such processes can be described by NB lineage distributions at all times of their evolution and are stable to the inclusion of additional event types [39, 40].

### Origin time vs TMRCA

The definition of the likelihood above assumes the origin of the phylogeny, *t*_0_ in Fig 1, is known or is a parameter to be estimated. This follows as we require the initial condition *M*_0_(0, *z*) = 1. In practice the phylogeny will likely only be known up to the time of the most recent common ancestor (TMRCA), *t*_1_ in Fig 1. We might account for this in one of two ways. The first, and simplest, is to treat the origin time as an additional parameter to be estimated. The second is to set a boundary condition at the TMRCA and to estimate the distribution of hidden lineages at that point, *H*_1_.

If one were confident the outbreak had stemmed from a single initial case, then the former method would be more suitable, especially if there was prior knowledge to constrain the time of origin. On the other hand, if we faced substantial uncertainty about how the outbreak began (ie there may have been numerous importations of the pathogen) and sequencing was sparse, (ie small *ψ* and *ρ*) then the TMRCA may be considerably more recent than the origin time and estimating the origin would be challenging. In this case, the latter approach may be more suitable. This would involve estimating the distribution of *H*_TMRCA_ and hence its GF *M*_1_(*t*_TMRCA_, *z*), from the family of NB distributions.

### Sources of error and bias

There are three primary sources of error in estimates generated using TimTam: there is approximation error, (ie the difference between the true likelihood and the TimTam approximation), there is the potential for estimator bias, since in general taking the mean or median of the posterior distribution for a finite sample is not guaranteed to give an unbiased estimate, and there is model misspecification due to differences in the data generating process and the birth-death process we are modelling it with.

Approximation error is the difference between the values computed using TimTam and the true values of the likelihood for the model described above. To assess the approximation error of this method we need to have access to the truth to compare it to. Assuming the error in an existing numeric method [32] is negligible, we can compare the values produced by each method to assess the approximation error.

We expect, based on the simulation results below, that our estimates are consistent, ie they will converge to their “true” values as the dataset size increases, however, they may be biased. We refer to the error in the estimates that would occur even if we had access to the exact values of the log-likelihood as the bias of the estimator. This is distinct from the error in the estimates due to the approximation of the log-likelihood (the approximation error).

Since we are simulating data from our model there is no model misspecification when we are analysing the unscheduled samples (ignoring the effects of the prior distribution). However, there is model misspecification when we aggregate unscheduled samples and treat them as scheduled samples. We carried out a simulation study to assess the accuracy of our estimates given these sources of error and bias.

## Results

### Model validation and computational complexity

To measure the approximation error and computational cost of TimTam we performed a simulation study comparing it to the method developed by Manceau *et al* [32], hereafter called the ODE approximation. The parameters used to generate a stratified set of simulations are given in Table 1 with an additional *ρ*-sample at the end of the simulation, after 35 days, with *ρ* = 0.5. These parameters were derived from estimates pertaining to SARS-CoV-2 [41] as described in S1 Appendix. A full description of the simulation and subsampling process used to generate these test data can also be found in S1 Appendix.

**Table 1:** Parameters used to simulate datasets. These parameters were derived from estimates pertaining to an outbreak of SARS-CoV-2 in Australia and are described in S1 Appendix. Rates are given in units of per day, the average duration of infectiousness is 10 days and the basic reproduction number is 1.85.

Parameter	Description	Value
λ	Birth rate	0.185
*μ*	Death rate	0.046
*ψ*	Sequenced sampling rate	0.008
*ω*	Unsequenced sampling rate	0.046

**Fig 2:**
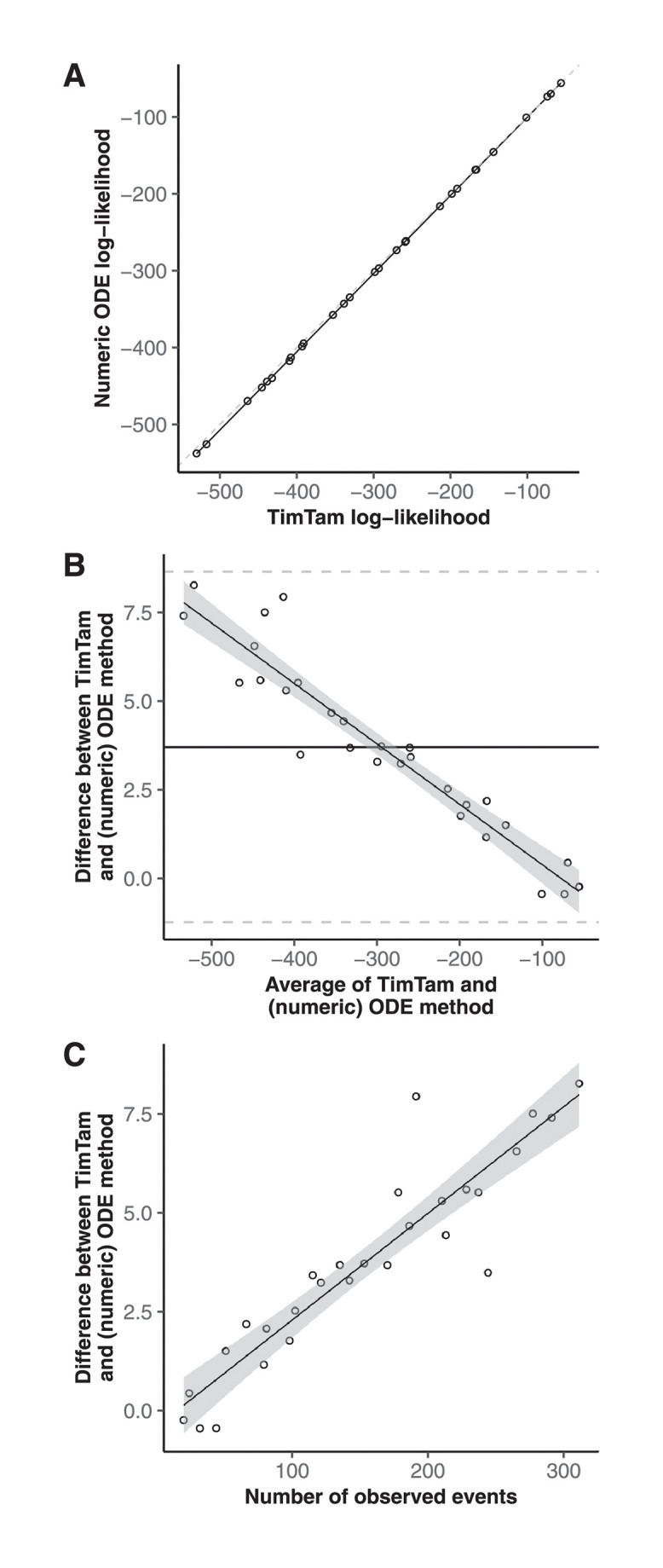
Likelihood comparison. TimTam tends to overestimate the log-likelihood on larger datasets, but this tendency is small relative to the overall variability in the log-likelihoods across the simulations. **(A)** The log-likelihood evaluated using TimTam and the ODE approximation are in good agreement. **(B)** A Bland-Altman plot comparing the values from TimTam and the ODE approximation reveals that there is a small systematic difference in the methods. **(C)** TimTam appears to overestimate the log-likelihood on larger datasets but the relative error is small.

Fig 2 shows the values of the log-likelihood function evaluated using both TimTam and the ODE approximation. The Bland-Altman plot shows that there is a systematic difference in the values, however this is small relative to the actual value of the log-likelihood.

**Fig 3:**
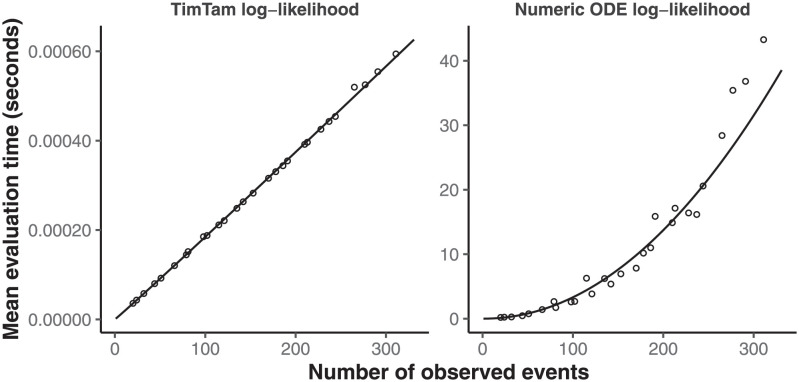
Log-likelihood evaluation time comparison. The time required to evaluate our approximation, TimTam, scales better with the dataset size than the existing ODE approximation. The scatter plots indicates the average number of seconds required to evaluate the log-likelihood function for each dataset size. The left panel contains the results using our approximation, which has times growing approximately linearly with the dataset size. The right panel contains the results using the ODE approximation, which has times growing approximately quadratically with the dataset size. Solid lines show least squares fits. Note that the *y*-axes are on different scales. The overall scaling factor (but not the exponent of the fitted model) may be implementation dependent.

To explore the computational complexity of TimTam, we measured how long it took to evaluate the log-likelihood for each of the simulated datasets. Fig 3 shows that with TimTam, the mean evaluation time grows approximately linearly with the size of the dataset, ∝ *n*^1.02^, where the 95% confidence interval (CI) on the exponent is (1.01, 1.03). In contrast, for the ODE approximation, the evaluation time grows approximately quadratically, ∝ *n*^2.05^, (95% CI = 1.94, 2.16). Since the ODE approximation requires specification of a truncation parameter, we obtained values for this parameter by increasing its value by 10 until doing so further resulted in a change to the log-likelihood of < 0.1%. The resulting truncation parameters are shown in Fig B in S1 Appendix. Full details of how the data were simulated, how the benchmarks were evaluated, and how the truncation parameter was selected are given in S1 Appendix.

In addition to the improvement in computational complexity, average evaluation times are orders of magnitude smaller for TimTam, which takes less than a millisecond in comparison to the seconds needed to evaluate the ODE approximation for larger datasets. We caution against over-interpreting the absolute computation times, since we implemented TimTam in Haskell whereas the implementation of the ODE approximation is a combination of C and Python [32]. However, to give some context these numbers we can consider the analyses performed by Vaughan *et al* [41]. The 15 outbreaks they considered had between 9 and 217 sequences with a median of 31 and they ran their MCMC chains for 10^8^ iterations. To evaluate this log-likelihood function 10^8^ times for a dataset with 31 sequences, using TimTam and the ODE approximation, the fitted models for the timing predict this would take approximately 3 hours and 17 months respectively.

### Parameter identifiability and aggregation scheme

Having validated TimTam against the ODE approximation, we now showcase our approach as an estimation scheme. We also explore the effect of aggregating unscheduled samples into scheduled sampling events. This allows us to assess the combined effect of the approximation error and estimator bias (in the case of the unscheduled samples) and the result of additional model misspecification when the data are aggregated at daily and weekly resolutions.

We simulated a dataset using the rate parameters in Table 1, ie a simulation which only contains unscheduled samples. The simulation was started with a single infectious individual and stopped at *t* = 50 days. From the unscheduled observations a second dataset was derived, this was done by aggregating the unscheduled observations into scheduled observations, eg all the unscheduled sequences sampled during the interval (*t*_*a*_, *t*_*b*_] were combined into a single scheduled sequenced sample at time *t*_*b*_ (as illustrated in Fig 1D). The sequenced samples were aggregated into daily observations and unsequenced samples were aggregated into weekly counts (with an offset of 12 hours to prevent sequenced and unsequenced samples occurring simultaneously).

**Fig 4:**
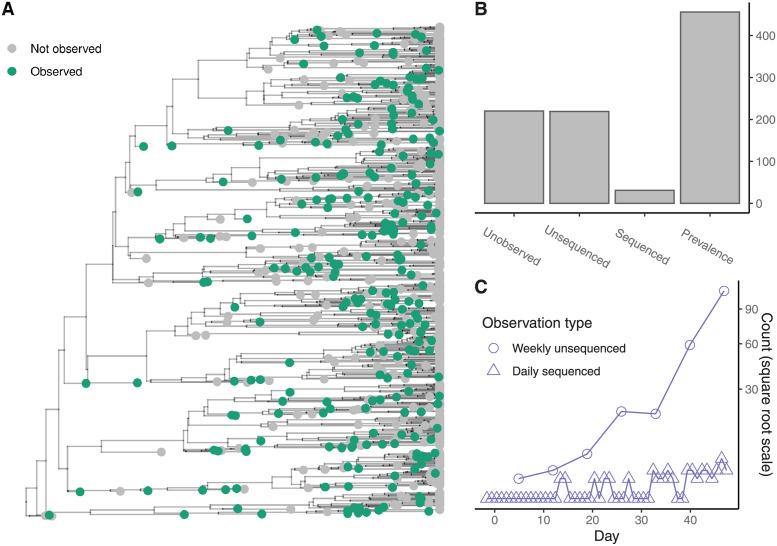
Simulation and aggregation. The tips of the transmission tree are subsampled to reflect the observation process. **(A)** The full transmission tree of the simulated epidemic where green tips have been observed either as sequenced or unsequenced samples. **(B)** Bar chart showing the number of unobserved infections, the number of observed and potentially sequenced infections and the prevalence at the end of the simulation. **(C)** Time series of the number of cases after aggregation: the sequenced samples are aggregated into daily counts and the unsequenced occurrences are aggregated into weekly counts. Fig 5 shows the marginal posterior distributions using either the raw or aggregated data above.

**Fig 5:**
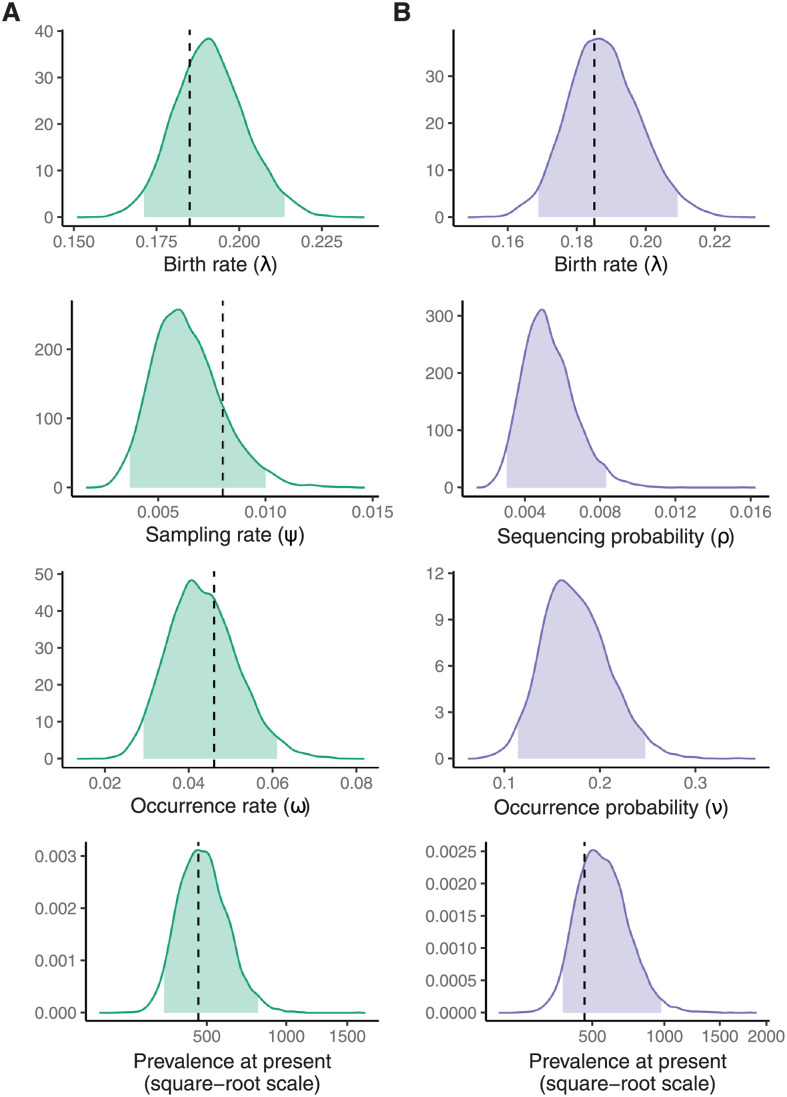
Posterior distributions. The marginal posterior distributions of the parameters and the prevalence at the end of the simulation given the death rate, *μ*. **(A)** The marginal posterior distributions using the simulation data shown in Fig 4. **(B)** The marginal posterior distributions using the aggregated simulation data. Filled areas indicate 95% credible intervals. Vertical dashed lines indicate true parameter values where they exist (Table 1). There are no vertical lines for the scheduled observation probabilities because they are not well defined for this simulation.

Fig 4A shows the sequenced and unsequenced samples as a subset of the whole simulation. Fig 4B shows the number of each type of outcome in the simulation along with the prevalence of infection when the simulation terminated. Fig 4C shows the same dataset after aggregation. Fig 5 shows the marginal posterior distributions of λ, and either *ψ* and *ω*, or *ρ* and *ν* depending on the dataset used.

When estimating model parameters the death rate *μ* was fixed to the true value used while simulating the data, since not fixing one of the parameters makes the likelihood unidentifiable and estimates of *μ* may be obtained from additional data sources [13, 42]. A uniform prior distribution was used for all parameters. The posterior samples where generated via MCMC. Standard diagnostics were used to test the convergence and mixing of the MCMC, (further details of the MCMC diagnostics and visualisations of the joint distribution of the posterior samples are given in S1 Appendix.).

### Repeated simulation to test credible interval coverage

**Fig 6:**
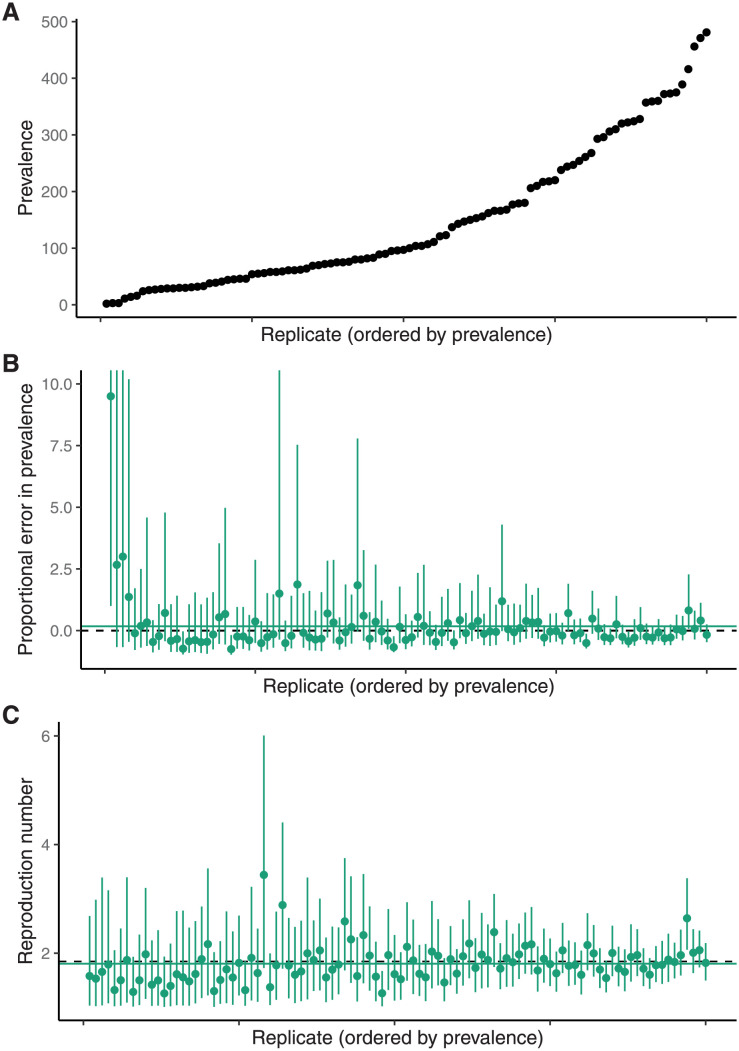
Simulation study results. The bias in the estimators of the basic reproduction number, $R_{0}$, and the prevalence is small and decreases with outbreak size. **(A)** The prevalence at the end of each of the simulations sorted into increasing order. **(B)** The proportional error in the prevalence estimate (ie a value of zero indicated by the dashed line corresponds to the true prevalence in that replicate). The solid green line is the mean of the point estimates. **(C)** The $R_{0}$ point estimates and 95% CI for each replicate. The solid green line is the mean of the point estimates. The corresponding intervals for other parameters using the aggregated data are shown in Figs F–I in S1 Appendix.

To test the calibration of the credible intervals (CIs) we performed a simulation study. Fig 6A shows prevalence at the end of 100 simulations generated using the same configuration as the single replicate described above.

Fig 6B shows the 95% CI and point estimate (posterior median) of the proportional error in the estimate of the prevalence in each replicate (ie the proportion by which the estimate differs from the true prevalence in that particular replicate; for an estimate $\hat{\theta }$ of *θ*, this is $(\hat{\theta }-\theta )/\theta$). The proportional error is used rather than the absolute error because the true prevalence varies substantially across replicates, making it difficult to compare them (for completeness we have included the raw prevalence and estimates in Fig G in S1 Appendix. In this figure the replicates in the top and bottom panels are in the same order. Of the 100 replicates, 92 have a CI containing the true prevalence at the end of the simulation (and hence contain 0). Fig 6C shows the 95% CI and point estimate (posterior median) of the basic reproduction number, $R_{0}=\lambda /(\mu +\psi +\omega )$, for each of 100 simulation replicates. Of the 100 replicates, 98 have a CI containing the true $R_{0}$. S1 Appendix contains some commentary on the level of coverage that is expected.

**Fig 7:**
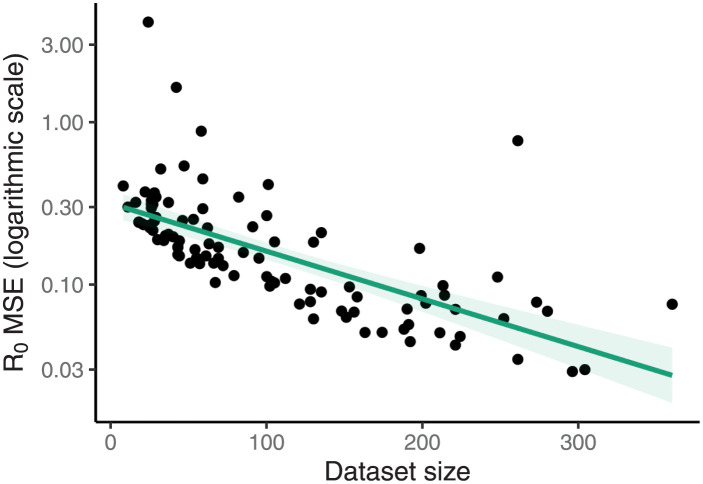
Mean squared error of estimates decreases with larger datasets. The mean squared error in the estimates of $R_{0}$ under the posterior distribution decreases as the size of the dataset increases. The corresponding figure looking at the estimates of the prevalence, using both scheduled and aggregated data, is given as Fig J in S1 Appendix.

Fig 7 shows the relationship between the mean-squared-error (MSE) in the estimates of $R_{0}$ under the posterior distribution and the size of the dataset used: the MSE decreases significantly with the size of the dataset. There is an analogous figure showing the MSE of the proportional error in the estimates of the prevalence using both the unscheduled samples and the aggregated values in given as Fig J in S1 Appendix.

Uniform prior distributions were used for all of the parameters. Analogous estimates were performed for the aggregated data (generated using the process described above). The estimates of the prevalence at the present are similarly unbiased for the aggregated data. Full results are presented in S1 Appendix.

## Discussion

We have described an analytic approximation, called TimTam, for the likelihood of a birth-death-sampling model which can also describe *scheduled data*, ie cohort sampling or reporting at predetermined times. TimTam can be used to analyse both sequenced and unsequenced samples, ie the observations can represent sequences that are either included in the reconstructed tree, or observed infections that are not sequenced (occurrence data). Our approach generalises previous birth-death estimation frameworks [29, 32, 33] by accommodating and exploiting more data types than previously considered and makes it feasible to analyse very large datasets.

Our work is a step towards more flexible, time series-based approaches to phylodynamics, in which multiple sequences are processed concurrently as elements of a time series. This extends the more common point-process based paradigm, in which samples are considered individually. TimTam also provides an estimate of the distribution of the prevalence of infection, allowing both the estimation of summary statistics, such as $R_{0}$, and the total number of cases. This approach does not admit a quantification of superspreading, although this would be an interesting extension to consider.

Comparison with an existing algorithm on small-to-moderate sized datasets suggests TimTam provides a good approximation of the true likelihood. While the approximation error appears to increase with the size of the dataset in absolute terms, this appears to be outweighed by the influence of additional data. Subsequent simulation studies demonstrate that the method can be used to obtain good estimates of the basic reproduction number and prevalence, and that the mean-squared error of these estimates decreases as the dataset gets bigger. The credible intervals associated with our estimates also appear to be well-calibrated.

We have presented the likelihood in terms of a known origin time, to simplify the presentation. As mentioned above, it is possible to express the likelihood in terms of the TMRCA instead. The distinction between these times, and the capacity to estimate either is important when studying the emergence of novel pathogens [43]. Further work is required to understand the capacity of this likelihood to estimate these quantities.

Based on existing results [24], we conjecture that if the probability of extinction becomes large, the zero inflation in the geometric distributions describing the number of descending lineages might become an issue. Since our focus is on large datasets describing established epidemics, we expect that this situation will rarely arise in practice. Additionally, as the death rate increases, the power of birth-death models as an inference tool is naturally limited by a lack of data [44, 45]. If this method is applied to small outbreaks or, when the basic reproduction number is low, sensitivity analyses will be necessary to check the fidelity of the negative binomial approximation.

Our work echoes existing frameworks [29, 32], but trades some generality for simplicity and tractability. Specifically, the particle filter method is more flexible [29] and the numerical schemes provide a complete posterior predictive distribution of prevalence through time, which allows the study of historical transmission [32]. Another limitation of our approach, which is common to many models, is to neglect *sampled ancestors*, ie individuals who have been observed but remain in the infectious population [32, 33, 46]. While the former can describe a greater variety of birth-death processes and the latter can be used to estimate additional properties of the process, the scalability of both frameworks are limited by their computational burden.

Our approximation provides a computationally efficient method for handling diverse data types (such as data aggregated to a daily or weekly resolution) that is scalable to large datasets. We also introduce an aggregation scheme that radically reduces the computational burden with only a modest expense to the accuracy. The improvement in performance stems from the resulting likelihood computation scaling with the number of aggregated intervals, proportional to epidemic duration, rather than the epidemic size. In many real epidemic scenarios data are only reported at a particular temporal resolution and in such scenarios this aggregation reflects the best-case for inference. As the availability of phylogenetic data (derived from sequences or contact-tracing) increases and the size of these data grows, such approximation schemes will become increasingly valuable.

## Supporting information

S1 Appendix Additional details of the approximation scheme and computational methodology. This document provides additional details regarding the derivation of the approximation scheme and provides additional detail on the selection of parameters for the simulation along with the simulation and benchmarking computations. **Fig A. Birth-death model of transmission and observation with scheduled samples**. In addition to unscheduled sampling which occurs continuously, we consider scheduled sampling where at predetermined times a binomial sample of the infectious population is removed. This corresponds to a cross-sectional study of prevalence. **(A)** The vertical lines indicate the timing of the scheduled samples: the dashed line (at time *t*_7_) is an unsequenced sample which observed two infectious individuals, the solid line (at time *t*_11_) is a sequenced sample. **(B)** The transmission tree corresponding to the realisation of the birth-death process, which appears in Panel A. **(C)** The reconstructed tree with sequenced observations on its leaves and the unsequenced observations as a point process. The example in this figure differs from Fig 1 of the main text in that here none of the unscheduled samples have been aggregated, the scheduled data has been generated as part of the observation process. **Fig B. The truncation parameter required by the ODE approximation grows approximately linearly with the size of the dataset**. Each point in the scatter plot shows the size of the truncation parameter for a simulated dataset. The solid line shows a linear least squares fit. **Fig C. The likelihood decreases approximately linearly with the size of the dataset**. The size of the simulated dataset and the associated likelihood (when calculated as a mean of the two methods considered). **Fig D. Posterior distribution conditioned upon unscheduled observations**. A scatter plot of samples from the posterior distribution showing their pairwise correlation. Given the death rate, *μ*, the posterior distribution given unscheduled observations has a well-defined maximum. **Fig E. Posterior distribution conditioned upon aggregated observations**. A scatter plot of samples from the posterior distribution showing their pairwise correlation. Given the death rate, *μ*, the posterior distribution (from aggregated unscheduled observations) has a well-defined maximum. **Fig F. The 95% range of proportional error in the estimates of the prevalence across the replicates**. The top panel shows the results using the unscheduled observations. The bottom panel shows the results when these unscheduled events are aggregated and treated as scheduled observations. The dashed line corresponds to zero error. The estimates are ordered by final prevalence in the simulation demonstrating that for larger outbreaks the proportional error is smaller. **Fig G. The 95% credible interval for the prevalence estimate and the true prevalence in that simulation**. The line segments show the credible interval and the black dots the true prevalence at the end of the simulation. The top panel shows the results using the unscheduled observations. The bottom panel shows the results when these unscheduled events are aggregated and treated as scheduled observations. **Fig H. Estimates of the birth, sampling and occurrence rates across the replicates using the simulated unscheduled observations**. The line segments show the 95% credible intervals for the estimates. The dashed horizontal lines indicate the true value of the rate used to simulate the data. **Fig I. Estimates of the birth rate, and sequenced and unsequenced sampling probabilities across the replicates using the aggregated observations**. The line segments show the 95% credible intervals for the estimates. The dashed horizontal lines indicate the true value of the rate used to simulate the data. There is no dashed line for the probabilities because they are not well-defined. **Fig J. The mean-squared-error in the estimate of the prevalence (as a proportion of the true prevalence) is smaller for larger datasets**. There is a point in this graph for each simulation used in the credible interval calibration example. The top panel shows the decreasing error using the unscheduled data and the bottom panel shows the decreasing error using the aggregated data.
(PDF)
