# Supplementary material for: A computationally tractable birth-death model that combines phylogenetic and epidemiological data
Source: PLoS Comput Biol. 2022 Feb 11;18(2):e1009805. doi: 10.1371/journal.pcbi.1009805 (PMC8903285; doi:10.1371/journal.pcbi.1009805)
Supplement: S1 Appendix — This document provides additional details regarding the derivation of the approximation scheme and provides additional detail on the selection of parameters for the simulation along with the simulation and benchmarking computations. Fig A. Birth-death model of transmission and observation with scheduled samples. In addition to unscheduled sampling which occurs continuously, we consider scheduled sampling where at predetermined times a binomial sample of the infectious population is removed. This corresponds to a cross-sectional study of prevalence. (A) The vertical lines indicate the timing of the scheduled samples: the dashed line (at time t7) is an unsequenced sample which observed two infectious individuals, the solid line (at time t11) is a sequenced sample. (B) The transmission tree corresponding to the realisation of the birth-death process, which appears in Panel A. (C) The reconstructed tree with sequenced observations on its leaves and the unsequenced observations as a point process. The example in this figure differs from Fig 1 of the main text in that here none of the unscheduled samples have been aggregated, the scheduled data has been generated as part of the observation process. Fig B. The truncation parameter required by the ODE approximation grows approximately linearly with the size of the dataset. Each point in the scatter plot shows the size of the truncation parameter for a simulated dataset. The solid line shows a linear least squares fit. Fig C. The likelihood decreases approximately linearly with the size of the dataset. The size of the simulated dataset and the associated likelihood (when calculated as a mean of the two methods considered). Fig D. Posterior distribution conditioned upon unscheduled observations. A scatter plot of samples from the posterior distribution showing their pairwise correlation. Given the death rate, μ, the posterior distribution given unscheduled observations has a well-defined maximum. Fig E. Posterior distribution [file pcbi.1009805.s001.pdf]

Appendix: A computationally tractable birth-death model that combines phylogenetic and epidemiological data

Alexander E. Zarebski*[1], Louis du Plessis[1], Kris V. Parag[2, †] and Oliver G. Pybus[1, †]

[1]Department of Zoology, University of Oxford
[2]MRC Centre for Global Infectious Disease Analysis, Imperial College London

[†]These authors contributed equally

> A generating function is a clothesline on which we hang up a sequence of numbers for display.
>
> Herbert S. Wilf, *Generatingfunctionology*

Theoretical results

Probability Generating Functions (PGF)

Recall that for a random variable, X, its probability generating function (PGF) in the variable z is

$$G_X(z) := \mathbb{E}_X\left[z^X\right].$$

Some useful elementary properties of G_X are that:

- $G_X(1^-) = 1$.
- $G'_X(1^-) = \mu$ where μ is the expected value of X.
- $\sigma^2 = G''_X(1^-) + G'_X(1^-) - (G'_X(1^-))^2$ where σ^2 is the variance of X.
- $G_S(z) = G_N(G_X(z))$ if $S = \sum_i^N X_i$ for IID X_i and has $G_{X_1+\cdots+X_n}(z) = G_X(z)^n$ as a special case.

Consequently, a generating function $H(z)$ which has positive coefficients with a finite sum, induces a distribution with PGF $G(z) := H(z)/H(1^-)$. In this case, we refer to $H(1^-)$ as the normalising constant.

Properties of the negative binomial distribution

Consider a negative binomially (NB) distributed random variable, $X \sim \text{NegBinom}(r, p)$. Its probability mass function (PMF) is:

$$\mathbb{P}(X = n) = \binom{n+r-1}{n}(1-p)^r p^n$$

The parameters r and p can be expressed in terms of the mean, μ, and variance, σ^2, as:

$$p = \frac{\sigma^2 - \mu}{\sigma^2} \quad \text{and} \quad r = \frac{\mu^2}{\sigma^2 - \mu}. \tag{1}$$

The probability generating function (PGF), $G(z; p, r)$ for this random variable is:

$$G(z; p, r) = \left(\frac{1-p}{1-pz}\right)^r \text{ where} \\ p = \frac{\sigma^2 - \mu}{\sigma^2} \quad \text{and} \quad r = \frac{\mu^2}{\sigma^2 - \mu}. \tag{2}$$

It is stated in the Methods section of the main text, if H has a negative binomial distribution, then conditioning on each of the observations leaves it with a negative binomial distribution. To see this, note both λ- and ψ-events do not influence H, hence conditioning upon them does not alter the distribution of H. Moreover, as the family of negative binomial PGFs is closed (up to a multiplicative constant) under both scaling of z and partial derivatives with respect to z, it can be shown by induction that:

$$\partial_z^n G(z; p, r) = r^{\bar{n}} \left(\frac{p}{1-p}\right)^n G(z; p, r+n),$$

where $x^{\bar{n}} := x(x+1)\ldots(x+n-1)$ is the rising factorial (a.k.a. the Pochhammer function). The corresponding result for scaling z is the following:

$$\begin{aligned} G(\alpha z; p, r) &= \left(\frac{1-p}{1-p\alpha}\right)^r \left(\frac{1-p\alpha}{1-p\alpha z}\right)^r \\ &= \left(\frac{1-p}{1-p\alpha}\right)^r G(z; p\alpha, r). \end{aligned}$$

*For correspondence email: alexander.zarebski@zoo.ox.ac.uk

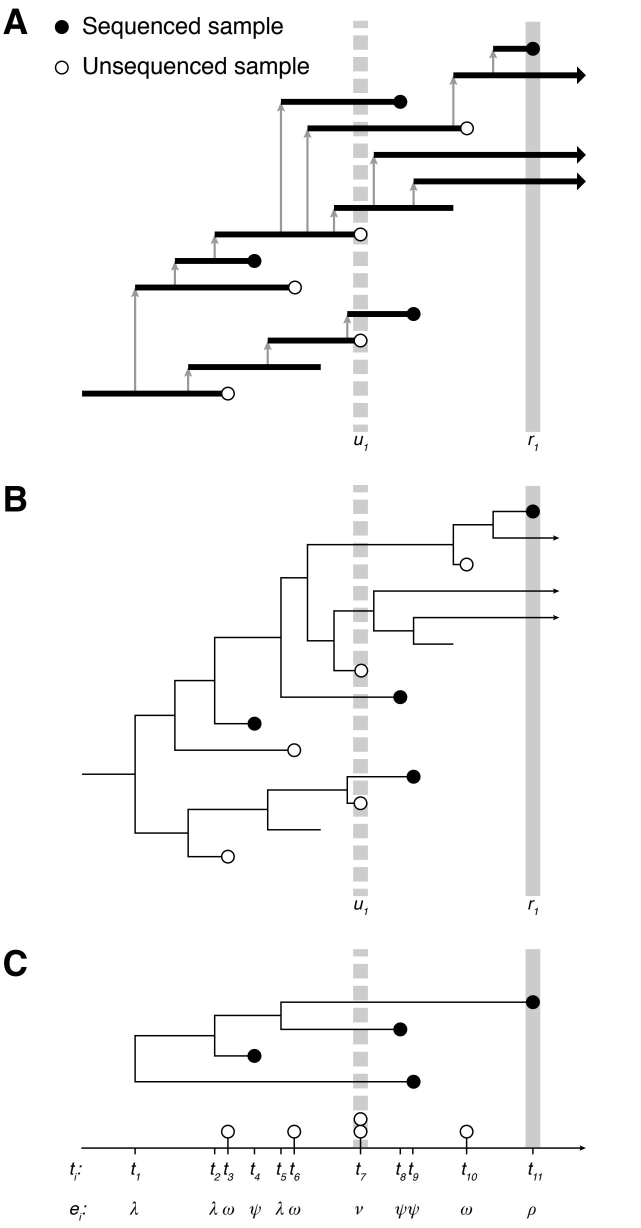

Fig A: Birth-death model of transmission and observation with scheduled samples. In addition to unscheduled sampling which occurs continuously, we consider scheduled sampling where at predetermined times a binomial sample of the infectious population is removed. This corresponds to a cross-sectional study of prevalence. **(A)** The vertical lines indicate the timing of the scheduled samples: the dashed line (at time t_7) is an unsequenced sample which observed two infectious individuals, the solid line (at time t_{11}) is a sequenced sample. **(B)** The transmission tree corresponding to the realisation of the birth-death process, which appears in Panel A. **(C)** The reconstructed tree with sequenced observations on its leaves and the unsequenced observations as a point process. The example in this figure differs from Fig 1 of the main text in that here none of the unscheduled samples have been aggregated, the scheduled data has been generated as part of the observation process.

These results are important for conditioning the distribution of H on both ρ- and ω-events.

Useful results for birth-death processes

Here we describe some results from the existing literature modified to match the notation and model we have adopted in the current work. Note that here we use forward-time in all equations, ie time is measured from the origin of the process to the present. First we have a couple of results adapted from **Theorem 3.1** of [1]. Consider the birth-death process (and notation) described in the Methods section of the main text, the probability that an individual, alive at time t, will generate no ψ-, ω- or ρ-sampled observations by time T when there is a ρ-sampling event. Let $p_0(u)$ denote this probability where $u := T - t$, then p_0 must satisfy the following differential equation:

$$p_0(0) = z \quad \text{and} \quad \frac{dp_0}{du} = \mu - \gamma p_0(u) + \lambda p_0(u)^2,$$

where the value of z will typically be $1 - \rho$ to denote the probability that the lineage was not ρ-sampled at time T. The solution is:

$$p_0(u, z) = \frac{x_1(x_2 - z) - x_2(x_1 - z)e^{-\sqrt{\Delta}u}}{(x_2 - z) - (x_1 - z)e^{-\sqrt{\Delta}u}} \qquad (3)$$

where

$$x_1 = \frac{\gamma - \sqrt{\Delta}}{2\lambda}, \quad x_2 = \frac{\gamma + \sqrt{\Delta}}{2\lambda}, \qquad (4)$$
$$\gamma = \lambda + \mu + \psi + \omega \quad \text{and} \quad \Delta = \gamma^2 - 4\lambda\mu.$$

In a similar manner, the probability of there being exactly one ρ-sampled lineage and no sampled extinct lineages, the function $p_1(u)$, satisfies the differential equation:

$$p_1(0) = 1 - z \quad \text{and}$$
$$\frac{dp_1}{du} = -\gamma p_1(u) + 2\lambda p_0(u) p_1(u). \qquad (5)$$

Note that for this equation, the initial condition is $1 - z$ since it will typically be used to indicate that the lineage was ρ-sampled at time T. Using the definitions in Equation (4), the solution is:

$$p_1(u, t) = \frac{(1 - z)\Delta}{\lambda^2} \frac{e^{-\sqrt{\Delta}u}}{((x_2 - z) - (x_1 - z)e^{-\sqrt{\Delta}u})^2}. \qquad (6)$$

These results were used in [2] to derive the generating function, $M(t, z)$, for the number of lineages that do not appear in a phylogeny during an interval of time without any observed events. The following result is adapted from **Proposition 4.1** of [2]. Consider the process described in the Methods section of the main text, during a period of time $[a, b]$ during which there are k lineages and no observed events, if the probability generating function for the number of lineages which do not appear in the phylogeny is initially $F(z)$, then the generating function satisfies the following PDE:

$$\begin{aligned} &M(a,z) = F(z) \quad \text{and} \\ &\quad \partial_t M = (\mu - \gamma z + \lambda z^2)\partial_z M + k(2\lambda z - \gamma)M \end{aligned} \tag{7}$$

The solution to this is:

$$M(t,z) = F(p_0(b-t,z))\left(\frac{p_1(b-t,z)}{1-z}\right)^k.$$

Unsequenced samples correspond to partial derivatives

First, we consider the case of *unscheduled*, unsequenced samples, which remove one H-lineage and occur with rate ω. Let $\mathcal{M}(z)$ be the generating function for the number of H-lineages prior to an observation. The j-th term of this series is $h_j z^j$, where h_j is the probability that the number of H-lineages is j. We want to find the corresponding term after one of the lineages has been removed at random. Since there are j lineages, there are j ways to sample one lineage, and upon sampling it is removed from the population (which then only has $j-1$ lineages.) Therefore, the term $h_j z^j$ becomes $\omega j h_j z^{j-1}$ (ie it forms the $(j-1)$-th term of the resulting generating function). Summing over j we find that the resulting generating function is equal to $\omega\partial_z\mathcal{M}(z)$. Selecting one of the lineages corresponds to the operation of *pointing* in combinatorics, [3], which would mean we take the partial derivative, ∂_z and then multiply by z. However, since we remove the lineage after selecting it, the results differ by a factor of z

For *scheduled* unsequenced samples, each H-lineage is sampled (and removed) with probability ν, or remains with probability $1-\nu$. Consider the case where ΔH of the H-lineages have been sampled. If there were j lineages to start with, the probability of sampling ΔH is:

$$\begin{aligned} &\binom{j}{\Delta H}\nu^{\Delta H}(1-\nu)^{j-\Delta H} \\ &= \frac{1}{(\Delta H)!}(j)_{\Delta H}\nu^{\Delta H}(1-\nu)^{j-\Delta H}, \end{aligned}$$

where $(x)_n = x(x-1)\dots(x-n+1)$ is the Pochhammer symbol. As above, we can then write down the terms of the generating function after the scheduled sampling event and sum over j to find the new generating function,

$$\frac{\nu^{\Delta H}}{(\Delta H)!}\partial_{\hat{z}}^{\Delta H}\mathcal{M}_i(\hat{z})|_{\hat{z}=(1-\nu)z}$$

where ∂_z^n indicates the n-th partial derivative.

Statistics of H via the generating functions M_i

The following equations describe the application of the properties above to the generating function for H. Consider the partial derivatives of M_i (which become relevant in the next section). Let $A = x_2 - x_1 e^{-\sqrt{\Delta}u}$, $B = 1 - e^{-\sqrt{\Delta}u}$ and $C = x_2 e^{-\sqrt{\Delta}u} - x_1$ in the expression for p_0 from Equation (3), we get the following form which simplifies some subsequent calculus:

$$\begin{aligned} p_0(u,z) &= \frac{x_1(x_2-z) - x_2(x_1-z)e^{-\sqrt{\Delta}u}}{(x_2-z)-(x_1-z)e^{-\sqrt{\Delta}u}} \\ &= \frac{x_1x_2(1-e^{-\sqrt{\Delta}u}) + (x_2e^{-\sqrt{\Delta}u} - x_1)z}{(x_2 - x_1e^{-\sqrt{\Delta}u}) - (1-e^{-\sqrt{\Delta}u})z} \\ &= \frac{x_1x_2B + Cz}{A - Bz} \end{aligned} \tag{8}$$

The expression for p_1 from Equation (6) can also be expressed in terms of A and B in a convenient form:

$$\begin{aligned} \frac{p_1(u,z)}{1-z} &= \frac{\Delta}{\lambda^2}\frac{e^{-\sqrt{\Delta}u}}{((x_2-z)-(x_1-z)e^{-\sqrt{\Delta}u})^2} \\ &= \frac{\Delta e^{-\sqrt{\Delta}u}}{\lambda^2}\frac{1}{(A-Bz)^2}, \end{aligned} \tag{9}$$

In subsequent calculations we will need the partial derivatives (with respect to z) for both p_0 and $p_1/(1-z)$ which are:

$$\begin{aligned} \partial_z p_0(u,z) &= \frac{CA + x_1x_2B^2}{(A-Bz)^2} \quad \text{and} \\ \partial_z^2 p_0(u,z) &= \frac{2B(CA + x_1x_2B^2)}{(A-Bz)^3}. \end{aligned} \tag{10}$$

and

$$\begin{aligned} \partial_z\left(\frac{p_1(u,z)}{1-z}\right) &= \frac{\Delta e^{-\sqrt{\Delta}u}}{\lambda^2}\frac{2B}{(A-Bz)^3} \quad \text{and} \\ \partial_z^2\left(\frac{p_1(u,z)}{1-z}\right) &= \frac{\Delta e^{-\sqrt{\Delta}u}}{\lambda^2}\frac{6B^2}{(A-Bz)^4}. \end{aligned} \tag{11}$$

We also have the following:

$$\begin{aligned} M(u,z) =& F(p_0(u,z))\left(\underbrace{\frac{p_1(u,z)}{1-z}}_{R(u,z)}\right)^k \\ \partial_z M(u,z) =& F'(p_0(u,t))\partial_z p_0(u,z)R(u,z)^k + \\ & F(p_0(u,z))kR(u,z)^{k-1}\partial_z R(u,z) \\ \partial_z^2 M(u,z) =& \\ & F''(p_0(u,z))(\partial_z p_0(u,z))^2 R(u,z)^k + \\ & F'(p_0(u,z))(\partial_z^2 p_0(u,z))R(u,z)^k + \\ & 2F'(p_0(u,z))(\partial_z p_0(u,z))kR(u,z)^{k-1}\partial_z R(u,z) + \\ & F(p_0(u,z))k(k-1)R(u,z)^{k-2}(\partial_z R(u,z))^2 + \\ & F(p_0(u,z))kR(u,z)^{k-1}\partial_z^2 R(u,z) \end{aligned} \tag{12}$$

Although Equation (12) appears messy, the number of expressions that need to be evaluated can be reduced using Equations 10 and 11.

Computational results

Simulation parameters

The parameters (in Table 1 of the main text) used to simulate datasets were chosen to be representative of estimates for the first SARS-CoV-2 epidemic wave in Australia. Following [4] we assume an infectious period of 10 days, and extract their mean estimate of R_0 for the Australian outbreak as 1.85 using Webplotdigitizer 4.5 [5]. To accurately represent the amount of unsequenced pathogen diversity, we set the (sequenced) sampling rate equal to the ratio of sequenced samples and cumulative case counts (with a 10 day offset to account for the incubation period and reporting delay) over the time-period analysed in [4] (until 11 March 2020), where we took cumulative case counts from [6]. This results in $\psi = 0.008 \approx 9/1071$. From the average duration of infectiousness we see that

$$\mu + \psi + \omega = \frac{1}{10}$$

when using days as our unit of time. From the equation of R_0 we have

$$R_0 = \frac{\lambda}{\mu + \psi + \omega} = 1.85.$$

Finally, since only genomic data was used in this analysis we allow for the possibility of occurrence data and, where necessary to facilitate comparison, scheduled samples. We assumed that half of the unsequenced samples were observed:

$$\frac{\omega}{\omega + \mu} = 0.5.$$

Solving the equations above gives us the parameter values listed in Table 1. When benchmarking against ODE approximation we added a single sequenced scheduled sample ($\rho = 0.5$) at the end of the simulation (at the 35 day mark) so the evaluation of the likelihood would require all relevant subexpressions to be evaluated. For the subsequent investigation of the coverage properties of the credible intervals we simulated data sets for a duration of 50 days. Both of these values are similar to the estimated outbreak durations reported by [4].

Simulation and selection of truncation parameter

To compare the computational cost of evaluating the ODE approximation, [2], with our TimTam approach, we simulated datasets of varying size and measured the time it took to evaluate the log-likelihood for these datasets using each algorithm. This also demonstrates the degree to which the two approximations agree on the value of the log-likelihood.

We simulated 10000 realisations of the birth-death process using the parameters shown in Table 1 of the main text. Each simulation was started with a single infectious individual and terminated at time $t = 35$, at which point there is a scheduled sequenced sampling event with probability $\rho = 0.5$. These simulations where then filtered to get a more uniform distribution of dataset sizes. This was done by selecting the first simulation which contained a number of events which fell in a variety of ranges: 1–10, 11–20, etc, up to 491–500. Any intervals that did not contain a simulation with one of these sizes was left empty. This filtering process left 38 simulated data sets.

The ODE approximation has a truncation parameter which needs to be set to a large value but for which no selection criterion has been provided. To select the truncation parameter, starting from a value of 10 we increased it in increments of 10 until a value was reached where an additional increment did not change the value of the log-likelihood by more than 0.1%. If no such value was reached below a threshold of 210 then this simuation was removed from the test set. The resulting truncation parameter selected for each simulation is shown in Fig B. The selected truncation parameter tends to grow linearly with the number of observations in the dataset.

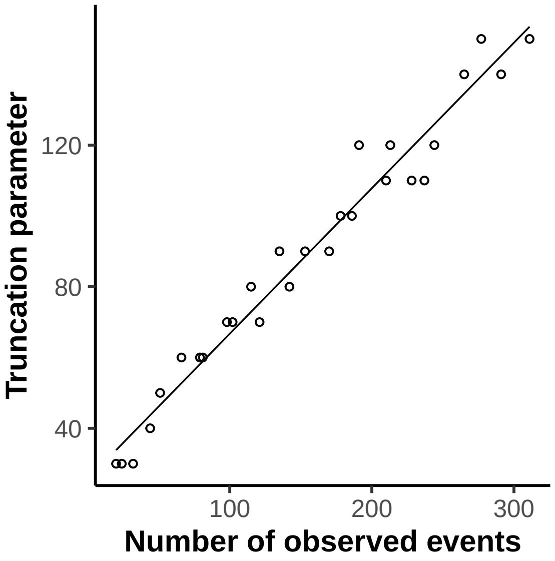

Fig B: The truncation parameter required by the ODE approximation grows approximately linearly with the size of the dataset. Each point in the scatter plot shows the size of the truncation parameter for a simulated dataset. The solid line shows a linear least squares fit.

Model validation and computational complexity

Fig C shows the relationship between the size of the data set considered and the average of the two log-likelihood calculations.

Our TimTam approximation was implemented in Haskell and the `criterion` library was used to estimate the average evaluation time (estimated by evaluating the log-likelihood for 5 seconds and counting the number of evaluations). For the ODE approximation we used the Cython implementation from [2] and used the Python Standard Library `timeit` module to estimate

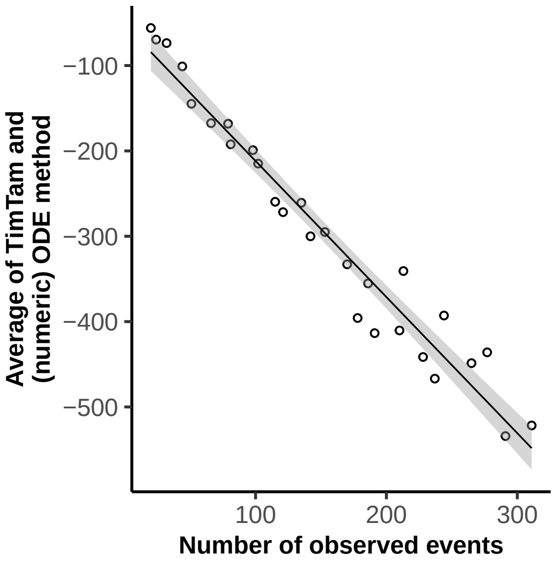

Fig C: The likelihood decreases approximately linearly with the size of the dataset. The size of the simulated dataset and the associated likelihood (when calculated as a mean of the two methods considered).

the average evaluation time (averaged over 10 replicates). We are most interested in the computational complexity of the algorithms in terms of the size of the dataset they are applied to. Because the implementations are in different languages, an absolute comparison is difficult (although since Cython and Haskell are used, they should give a reasonable indication of the performance that could be achieved with a low-level language).

To model the computational complexity of the likelihoods we fit a linear model to the logarithms of the evaluation times and the size of the dataset. Thus, if n is the size of the dataset, the time to evaluate the log-likelihood, t_{eval}, we have $t_{\text{eval}} \propto n^a$. An estimate of $a \approx 1$ suggests a linear complexity and an estimate ≈ 2 a quadratic complexity. The average evaluation times and the model fit are shown in Fig 3 of the main text.

Using TimTam, the estimated value of the exponent of the fitted model is 1.02 with a 95% confidence interval of $(1.01, 1.03)$. Using the ODE approximation it is 2.05 with a 95% confidence interval of $(1.94, 2.16)$. For both algorithms smaller datasets appear as outliers (likely due to the computational overhead of the programs.) We repeated the estimation process with robust linear regression (using `rlm` from the `MASS` package in R), under this model the exponent was 1.02, $(1.01, 1.02)$ for the TimTam likelihood and 2.05, $(1.94, 2.17)$ for the ODE approximation.

Parameter identifiability and aggregation scheme

In the simulation study of the effects of aggregation of observations described in the main text we used MCMC to characterise the posterior distribution. For each dataset a single chain was run for 5.001×10^6 iterations using a Gaussian kernel with a standard deviation of 0.01. The first thousand samples were removed as burn-in and the samples were thinned by a factor of 1000 leaving 5000 samples. Convergence was assessed via visual inspection of the traces. The effective sample size was greater than 200; diagnostics were computed using `coda` [7].

The joint distribution of the posterior samples conditional upon the unscheduled dataset are shown in Fig D and for the scheduled dataset obtained via aggregation in Fig E.

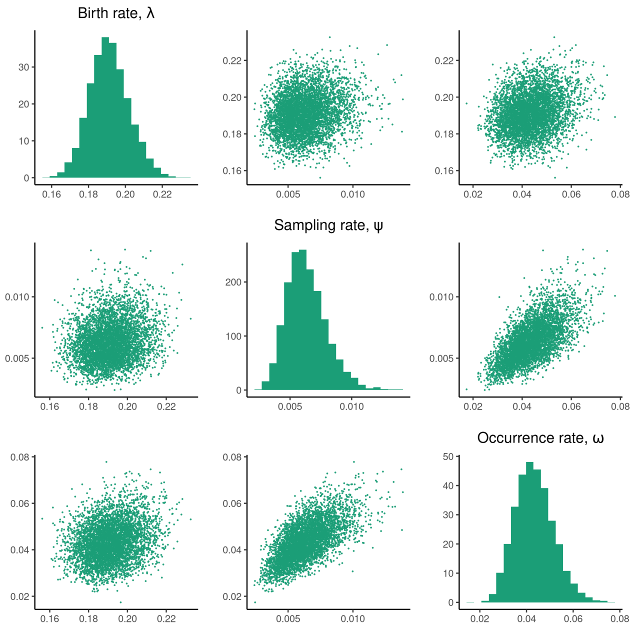

Fig D: Posterior distribution conditioned upon unscheduled observations. A scatter plot of samples from the posterior distribution showing their pairwise correlation. Given the death rate, μ, the posterior distribution given unscheduled observations has a well-defined maximum.

Repeated simulation to test credible interval coverage

A simulation study was carried out to test whether the 95% credible intervals (CI) of the birth rate, λ, and the prevalence at the present, $H(t_N)$, contain the values from the simulation. Since these are *credible intervals* (not confidence intervals) in general they will not contain the simulation parameters with the correct frequency. However, since we have used a uniform prior over the full support of the parameters, the posterior is proportional to the likelihood. In a large-sample setting, we would expect the CI to behave similarly to a confidence interval and contain the simulation parameters with approximately the correct frequency. The birth rate is used instead of the reproduction number because it is ill-defined in the case of the aggregated data. This is because there the scheduled events arise from post processing of the data, ie the binning of samples, instead of being generated by the sampling model.

Fig F shows 95% CI for the proportional error in the estimate of the prevalence at the present. Of 100 replicates, 92 and 89 of the CIs contained the true preva-

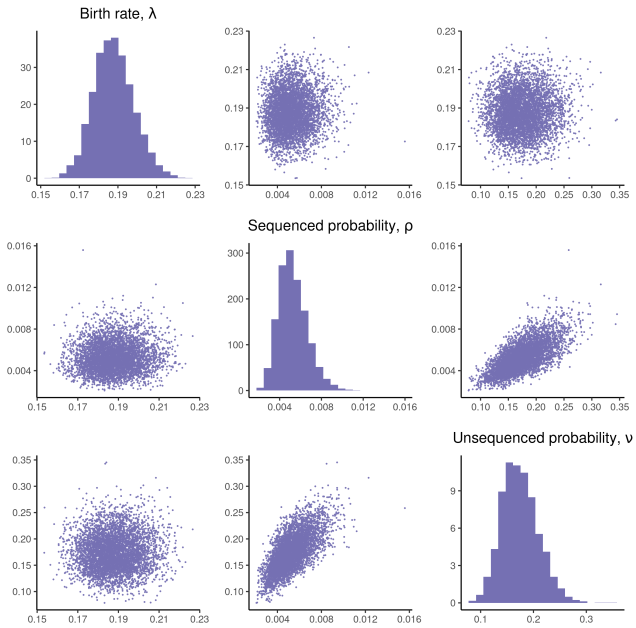

Fig E: Posterior distribution conditioned upon aggregated observations. A scatter plot of samples from the posterior distribution showing their pairwise correlation. Given the death rate, μ, the posterior distribution (from aggregated unscheduled observations) has a well-defined maximum.

lence from the simulation using the unscheduled and aggregated data respectively. Fig G shows the 95% CI for the estimate of the prevalence and the true prevalence in the simulation using either the unscheduled or aggregated data. Figs H and I show the 95% credible intervals for the estimate of the parameters. Of the 100 replicates 95 and 99 of the CIs contained the true birth rate. For the unscheduled data, the sampling rate and occurrence rate where contained in 94 and 97 of the CIs respectively.

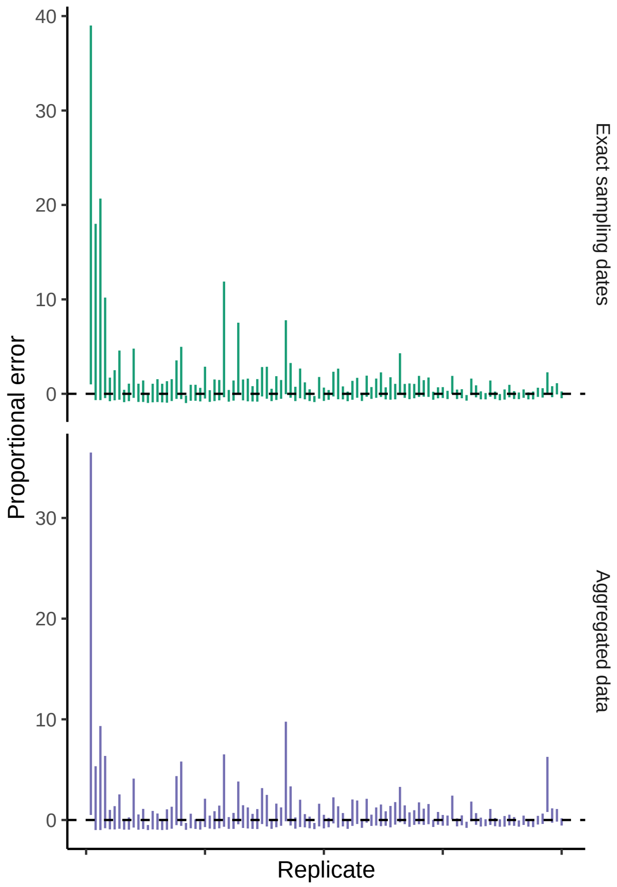

Fig F: The 95% range of proportional error in the estimates of the prevalence across the replicates. The top panel shows the results using the unscheduled observations. The bottom panel shows the results when these unscheduled events are aggregated and treated as scheduled observations. The dashed line corresponds to zero error. The estimates are ordered by final prevalence in the simulation demonstrating that for larger outbreaks the proportional error is smaller.

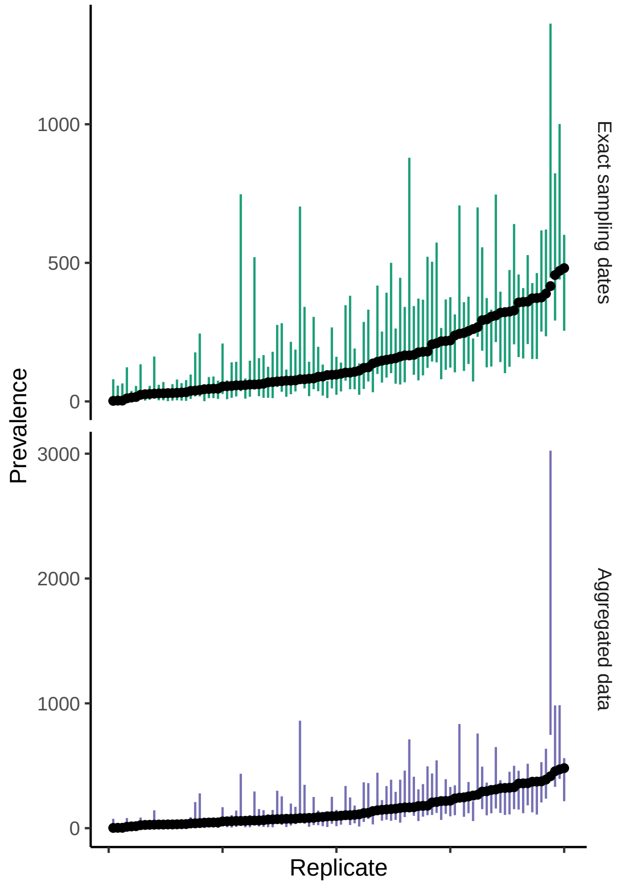

Fig G: The 95% credible interval for the prevalence estimate and the true prevalence in that simulation. The line segments show the credible interval and the black dots the true prevalence at the end of the simulation. The top panel shows the results using the unscheduled observations. The bottom panel shows the results when these unscheduled events are aggregated and treated as scheduled observations.

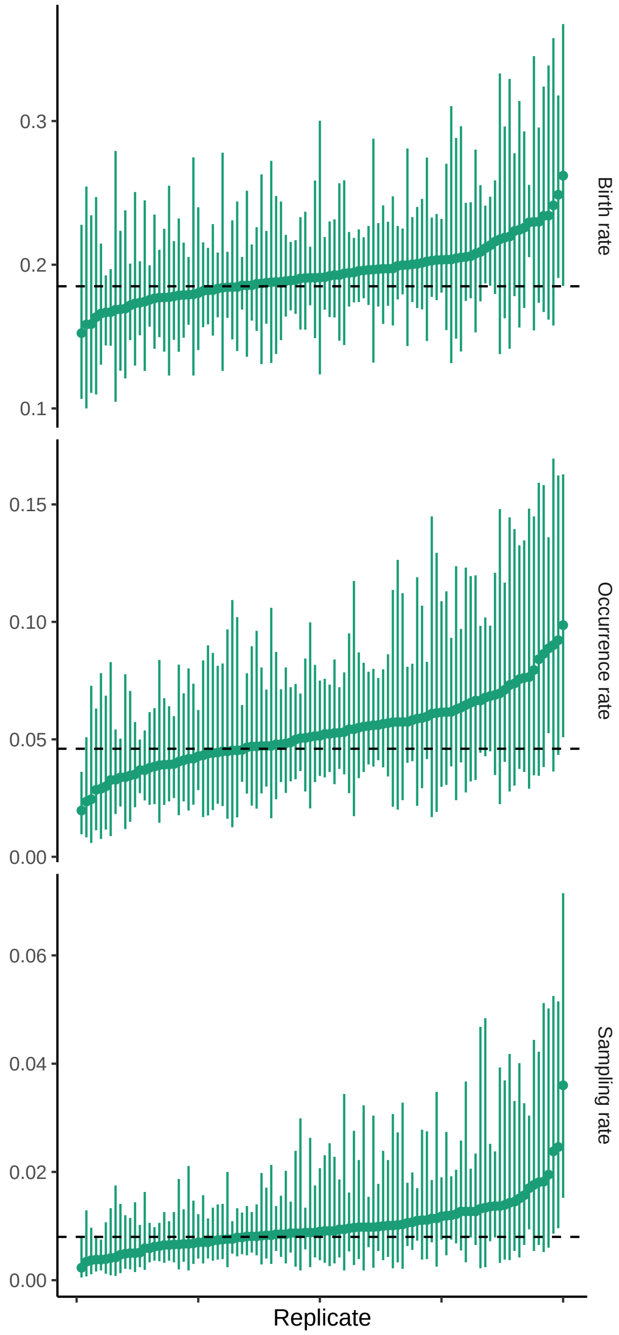

Fig H: Estimates of the birth, sampling and occurrence rates across the replicates using the simulated unscheduled observations. The line segments show the 95% credible intervals for the estimates. The dashed horizontal lines indicate the true value of the rate used to simulate the data.

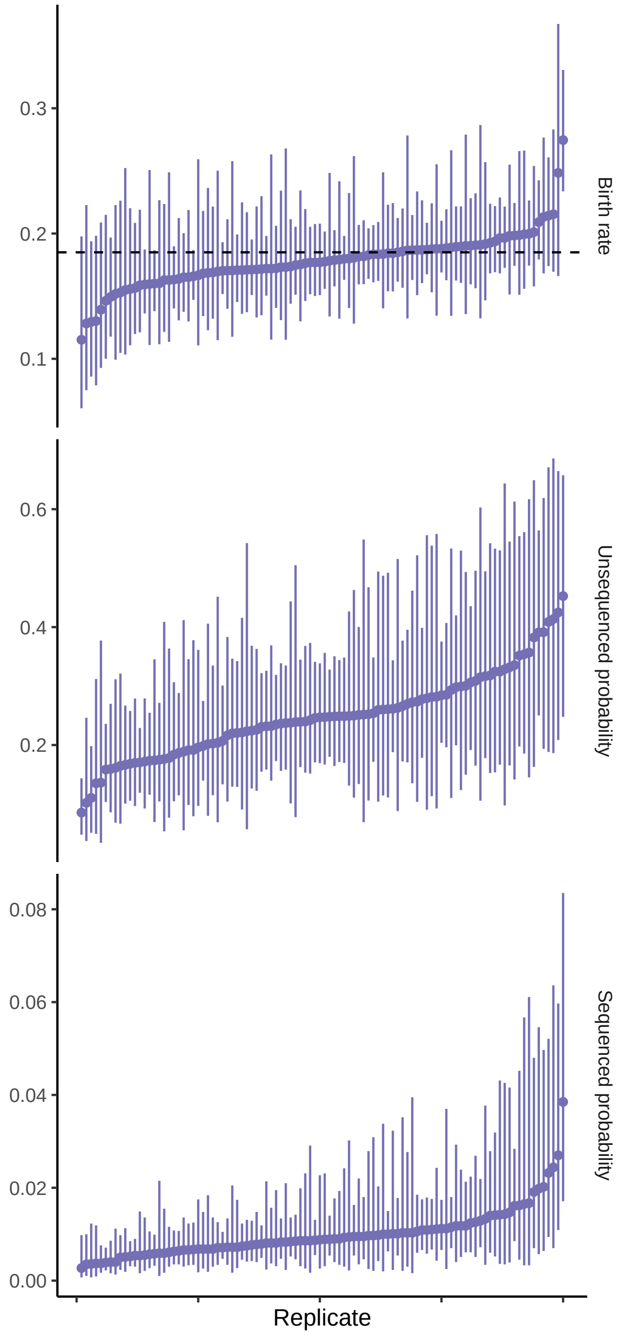

Fig I: Estimates of the birth rate, and sequenced and unsequenced sampling probabilities across the replicates using the aggregated observations. The line segments show the 95% credible intervals for the estimates. The dashed horizontal lines indicate the true value of the rate used to simulate the data. There is no dashed line for the probabilities because they are not well-defined.

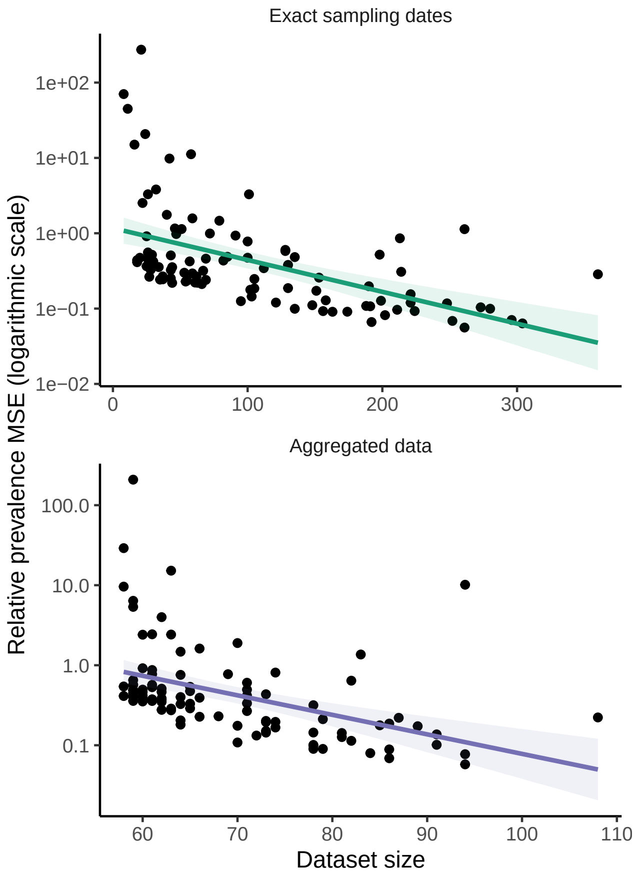

Fig J: The mean-squared-error in the estimate of the prevalence (as a proportion of the true prevalence) is smaller for larger datasets. There is a point in this graph for each simulation used in the credible interval calibration example. The top panel shows the decreasing error using the unscheduled data and the bottom panel shows the decreasing error using the aggregated data.

References

[1] Stadler T. Sampling-through-time in birth-death trees. Journal of Theoretical Biology. 2010;267(3):396–404. doi:10.1016/j.jtbi.2010.09.010.

[2] Manceau M, Gupta A, Vaughan T, Stadler T. The probability distribution of the ancestral population size conditioned on the reconstructed phylogenetic tree with occurrence data. Journal of Theoretical Biology. 2021;509:110400. doi:10.1016/j.jtbi.2020.110400.

[3] Flajolet P, Sedgewick R. Analytic Combinatorics. Cambridge University Press; 2009.

[4] Vaughan TG, Sciré J, Nadeau SA, Stadler T. Estimates of outbreak-specific SARS-CoV-2 epidemiological parameters from genomic data. medRxiv. 2020;doi:10.1101/2020.09.12.20193284.

[5] Rohatgi A. Webplotdigitizer: Version 4.5; 2021. Available from: https://automeris.io/WebPlotDigitizer.

[6] Dong E, Du H, Gardner L. An interactive web-based dashboard to track COVID-19 in real time. The Lancet Infectious Diseases. 2020;20(5):533–534. doi:10.1016/S1473-3099(20)30120-1.

[7] Plummer M, Best N, Cowles K, Vines K. CODA: Convergence Diagnosis and Output Analysis for MCMC. R News. 2006;6(1):7–11.
